# Identification and structure solution of fragment hits against kinetoplastid *N*-myristoyltransferase

**DOI:** 10.1107/S2053230X15003040

**Published:** 2015-04-21

**Authors:** David A. Robinson, Paul G. Wyatt

**Affiliations:** a Drug Discovery Unit, College of Life Sciences, University of Dundee, Dow Street, Dundee DD1 5EH, Scotland

**Keywords:** *N*-myristoyltransferase, *Trypanosoma brucei*, African trypanosomiasis

## Abstract

*Trypanosoma brucei* *N*-myristoyltransferase (*Tb*NMT) is an attractive therapeutic target for the treatment of human African trypanosomiasis. Pyrazole sulfonamide (DDD85646), a potent inhibitor of *Tb*NMT, has been identified in previous studies; however, poor central nervous system exposure restricts its use to the haemolymphatic form (stage 1) of the disease. In order to identify new chemical matter, a fragment screen was carried out by ligand-observed NMR spectroscopy, identifying hits that occupy the DDD85646 binding site. Crystal structures of hits from this assay have been obtained in complex with the closely related NMT from *Leishmania major*, providing a structural starting point for the evolution of novel chemical matter.

## Abstract 2

*N*-Myristoyltransferase (NMT) has been shown to be an attractive target for the development of novel therapeutic agents for the treatment of human African trypanosomiasis. A fragment library has been screened using NMR spectroscopy and the binding mode of the hits was confirmed by X-ray crystallography using *L. major* NMT as a structural surrogate.

## 1. Introduction   

Human African trypanosomiasis (HAT) is caused by two subspecies of the protozoan parasite *Trypanosoma brucei*, *T. b. gambiense* and *T. b. rhodesiense*, transmitted by the bite of an infected tsetse fly (Brun *et al.*, 2011 ▶; Stuart *et al.*, 2008 ▶). The disease is often fatal unless treated. It has two stages: an initial haemolymphatic infection during which the parasites are found in the bloodstream, which gives rise to nonspecific symptoms, and a second stage during which the parasites enter the central nervous system (CNS), giving rise to the classic symptoms of HAT, eventually leading to coma and death. Currently there are five treatments available, although none of them are satisfactory owing to toxicity, treatment failures and the requirement for parenteral administration, which is inappropriate in a rural African setting (Jacobs *et al.*, 2011 ▶).

**Figure 1:**
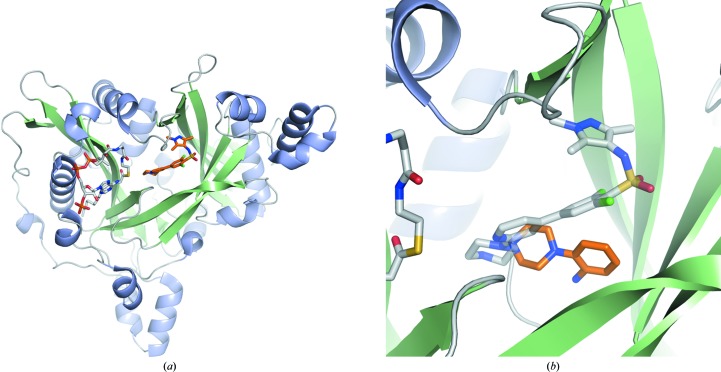
(*a*) *Lm*NMT in cartoon representation bound to the cofactor myristoyl-CoA (C atoms in grey) and the ligand DDD85646 (C atoms in gold; PDB entry 2wsa; Frearson *et al.*, 2010 ▶). (*b*) shows the orientation of DDD85646 (C atoms in grey) and a fragment hit (C atoms in gold) within the peptide-binding pocket of *Lm*NMT

*N*-Myristoyltransferase (NMT) catalyses the co-translational transfer of myristate from myristoyl-CoA to the N-terminal glycine of a large number of proteins, a modification that has been implicated in localization and/or activation of the substrate (Farazi *et al.*, 2001 ▶; Resh, 2006 ▶). The enzyme operates *via* a bi-bi mechanism in which it first binds myristoyl-CoA, causing a conformational rearrangement which subsequently reveals the peptide-binding site (Rudnick *et al.*, 1991 ▶). In *T. brucei*, RNAi knockdown of NMT has been shown to be lethal in cell culture (Price *et al.*, 2003 ▶) and to abrogate infectivity in animal models of HAT (Price *et al.*, 2010 ▶). Bioinformatics analysis suggests that about 60 proteins are myristoylated in the parasite (Bowyer *et al.*, 2008 ▶), although there is incomplete knowledge of the downstream targets (Price *et al.*, 2007 ▶). NMT has also been investigated as a potential target for the treatment of other parasitic diseases, including malaria (Wright *et al.*, 2014 ▶), leishmaniasis (Tate *et al.*, 2014 ▶) and Chagas disease (Roberts *et al.*, 2014 ▶). Previously, we have reported the development of the potent NMT inhibitor DDD85646 (compound **1**; Frearson *et al.*, 2010 ▶), a molecule that binds to the peptide-binding site (Fig. 1 ▶). It was also shown to be an excellent lead for drug development; however, a lack of CNS exposure resulted in poor efficacy against the CNS stage of the disease. New studies were initiated with the aim of identifying new chemical matter to act as start points, or modifications of existing series, to develop molecules with more attractive pharmacokinetic profiles.

Fragment-based lead discovery (FBLD) is now established as a validated technique to identify small, low-affinity molecules that act as starting points for the evolution of larger, higher affinity molecules (Hubbard & Murray, 2011 ▶). Owing to the low affinities of fragment hits towards their target (typically in the millimolar range), biophysical techniques are employed to detect ligand binding in place of conventional biochemical assays. NMR spectroscopy formed the basis of the first forays into FBLD using protein-observed methods to detect ligand binding (Shuker *et al.*, 1996 ▶). As the field has matured, ligand-observed NMR methods, observing changes in the NMR spectra of small molecules in the presence of a macromolecular target, have become an established workhorse technique for hit identification (Harner *et al.*, 2013 ▶).

Owing to the low affinity and limited complexity of fragment molecules, the majority of successful FBLD campaigns require structural biology, in the vast majority X-ray crystallography, to determine the binding modes of hit molecules to the target of interest.

## 2. Materials and methods   

### 2.1. Protein expression and purification   

The gene encoding *Tb*NMT was cloned into a pET-15-derived expression vector encoding an N-terminal 6×His tag followed by a TEV cleavage site and expressed in *Escherichia coli* Rosetta (DE3) cells using autoinduction medium. The cells were harvested by centrifugation and resuspended in 50 m*M* Tris–HCl, 300 m*M* NaCl, 20 m*M* imidazole pH 9.0 supplemented with 40% sucrose. DNase, lysozyme and a protease-inhibitor cocktail tablet were added before passage through a cell disruptor (Constant Cell Systems). Cleared lysate was prepared by centrifugation (50 000*g*, 30 min) before application onto a 5 ml HisTrap column (GE Healthcare) pre-equilibrated with 25 m*M* sodium phosphate, 0.3 *M* NaCl, 5 m*M* imidazole pH 8.0. *Tb*NMT was eluted with a 5–250 m*M* imidazole gradient and the fractions were pooled, diluted in 25 m*M* sodium phosphate pH 8.0 until the NaCl concentration was below 30 m*M* and applied onto a 6 ml Resource Q column (GE Healthcare) equilibrated with 25 m*M* sodium phosphate pH 8.0. Protein was eluted by the application of a 0–500 m*M* NaCl gradient.

*Lm*NMT (5–421) was cloned into a pET-15b-derived vector encoding an N-terminal 6×His tag followed by a TEV cleavage site and expressed in *E. coli* Rosetta (DE3) cells using autoinduction medium. The cells were harvested by centrifugation, resuspended in 50 m*M* HEPES pH 7.5, 0.5 *M* NaCl, 5 m*M* imidazole, 5% glycerol (plus DNase, lysozyme and a protease-inhibitor cocktail tablet) and lysed by passage through a cell disruptor (Constant Cell Systems). The lysate was cleared by centrifugation (50 000*g*, 30 min) and loaded onto a 5 ml HisTrap crude column (GE Healthcare), and the target protein was eluted in a 5–250 m*M* imidazole gradient. Fractions containing *Lm*NMT were pooled, desalted and applied onto a 6 ml Resource-Q column pre-equilibrated with 10 m*M* HEPES pH 7.5. *Lm*NMT was eluted with a 0–500 m*M* NaCl gradient and fractions were analyzed by SDS–PAGE; fractions with greater than 95% purity were concentrated to 9 mg ml^−1^ for crystallization. All chromatographic steps were carried out using an ÄKTA system (GE Healthcare)

### 2.2. Fragment screening by ligand-observed NMR spectroscopy   

*Tb*NMT was prepared at 10 µ*M* in 25 m*M* sodium phosphate pH 7.4 and 10% deuterated water and the cofactor myristoyl-CoA was added to a final concentration of 50 µ*M*. A fragment library composed of 652 molecules was screened in pools of eight molecules per sample, each at a concentration of 500 µ*M*. A suite of one-dimensional NMR experiments were carried out on each sample to identify fragments that interact with the protein: saturation transfer difference (STD; Mayer & Meyer, 1999*a* ▶,*b* ▶), water ligand observed by gradient (water-LOGSY; Dalvit *et al.*, 2000 ▶) and *T*_2_ relaxation-filtered one-dimensional NMR spectroscopy (Hajduk *et al.*, 1997 ▶), specifically using the Carr–Purcell–Meiboom–Gill (CPMG) sequence (Meiboom & Gill, 1958 ▶). The known competitor ligand DDD85646 was added to each sample to a final concentration of 50 µ*M* and the suite of NMR experiments was repeated to identify fragment molecules that no longer interact in the presence of the competitor, suggesting that they bind in a similar site to DDD85646. All NMR experiments were conducted at 298 K using a Bruker 500 MHz spectrometer equipped with a Txi cryoprobe; data were processed and visualized using *TopSpin* (Bruker).

### 2.3. Crystallization of *Lm*NMT   

To obtain crystals of *Lm*NMT complexed with the cofactor myristoyl-CoA (MCoA), *Lm*NMT (5–421) was prepared at 9 mg ml^−1^ in 10 m*M* HEPES pH 7.5, 0.5 *M* NaCl and the cofactor myristoyl CoA was added to a final concentration of 1 m*M*. Crystallization was carried out by the hanging-drop vapour-diffusion method in 24-well Linbro plates (Hampton Research). Drops were prepared by mixing 2 µl protein solution with 2 µl reservoir solution consisting of 24–30% PEG 1500, 0.2 *M* NaCl, 0.1 *M* sodium citrate pH 5.6. Rod-shaped crystals appeared after 2–4 d incubation at 293 K. The quality of the *Lm*NMT–MCoA crystals obtained using this method was variable and clusters were often formed; therefore, macro-seeding was employed to obtain single crystals. A cluster of crystals was crushed and transferred into 100 µl stabilization solution consisting of 35% PEG 1500, 0.2 *M* NaCl, 0.1 *M* sodium citrate pH 5.6, which was vortexed using a Seed Bead kit (Hampton Research) to create a homogenous solution of seed stock. In preparation for crystallization, this solution was diluted 1:10 with stabilization solution and a mounting loop was passed through the seed solution and streaked through the crystallization drop. This resulted in a larger number of single rod-shaped crystals that were suitable for further use. Protein–ligand complexes were obtained by soaking the crystals for 16 h in mother liquor-derived cryoprotectant (25% PEG 1500, 0.2 *M* NaCl, 0.1 *M* sodium citrate pH 5.6, 20% glycerol) with 15 m*M* ligand prepared from a stock concentration of 0.2 *M* in DMSO. Crystals were flash-cooled in liquid nitrogen in preparation for data measurement.

### 2.4. Data measurement and structure determination   

**Figure 2:**
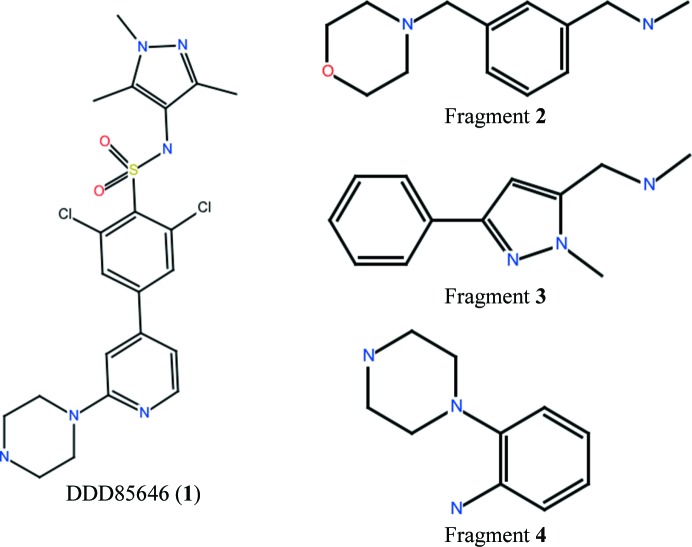
Small-molecule ligands described in this study: DDD85646, fragment **2**, fragment **3** and fragment **4**

Data sets were measured on beamline ID14-4 at the European Synchrotron Radiation Facility (ESRF) using an ADSC Q315 CCD detector for *Lm*NMT–fragment **2** and *Lm*NMT–fragment **3** and on beamline I04-1 at Diamond Light Source (DLS) using a Pilatus 2M detector for *Lm*NMT–fragment **4**. For all data sets integration was carried out using *XDS* (Kabsch, 2010 ▶) and data were reduced using *SCALA* (Evans, 2006 ▶) as implemented in automated pipeline data processing at the beamline using *xia*2 (Winter *et al.*, 2013 ▶). Phasing was carried out by molecular replacement as implemented in *MOLREP* (Vagin & Teplyakov, 2010 ▶) using the binary *Lm*NMT–MCoA complex (PDB 3h5z; Frearson *et al.*, 2010 ▶) as a search model, refinement was carried out using *REFMAC*5 (Murshudov *et al.*, 2011 ▶) and manual model alteration was carried out using *Coot* (Emsley & Cowtan, 2004 ▶). Ligand-coordinate and restraint files were generated using *PRODRG* (Schüttelkopf & van Aalten, 2004 ▶) and ligands were modelled into unbiased *F*_obs_ − *F*_calc_ density maps using *Coot*. The chemical structures of the small molecules used in this study are shown in Fig. 2 ▶. Data-measurement and model-refinement statistics are presented in Table 1 ▶. Coordinate files and associated experimental data have been deposited in the Protein Data Bank (PDB) with accession codes 4ucp, 4ucm and 4ucn for *Lm*NMT–fragment **2**, *Lm*NMT–fragment **3** and *Lm*NMT–fragment **4**, respectively.

### 2.5. Biochemical enzyme-inhibition assay   

Activity assays for *Tb*NMT and *Lm*NMT were carried out using a radiometric SPA-based assay as described previously (Frearson *et al.*, 2010 ▶; Brand *et al.*, 2014 ▶). Percentage inhibition (PI) was determined using a fragment concentration of 0.5 m*M* in the assay.

### 2.6. Biolayer interferometry   

*Tb*NMT and *Lm*NMT were biotinylated by incubation with NHS-PEG4-biotin (Thermo) in a 1:1 molar ratio for 30 min at room temperature before free biotinylation reagent was removed by passage through a 2 ml Zeba desalt spin column (Thermo). Biolayer interferometry (BLI) measurements were carried out using an Octet RED 384 instrument (ForteBio). *Tb*NMT and *Lm*NMT were immobilized upon superstreptavidin (SSA) biosensors by incubation for 900 s at 25 and 50 µg ml^−1^, respectively, before free streptavidin sites were blocked by a 60 s dip into 10 µg ml^−1^ biocytin (Tocris). A control set of SSA biosensors were prepared in parallel by blocking the surface with biocytin. A seven-point concentration series was prepared for each fragment molecule in threefold dilution steps from a top concentration of 1 m*M*. For each set of biosensors, a 60 s baseline in buffer alone was acquired followed by a 60 s association step and a 180 s dissociation step. All experiments were carried out using 25 m*M* HEPES pH 7.5, 150 m*M* NaCl as the buffer at 298 K. Binding experiments were repeated in the presence of 1 µ*M* myristoyl-CoA (Sigma). Data were processed and analysed and *K*_d_ values were determined using the global fitting procedures as implemented in *ForteBio Data Analysis Software* v.7.0.1.5. The ligand efficiency metric was calculated using LE = −*RT*ln*K*_d_/heavy-atom count (Hopkins *et al.*, 2004 ▶). The results are shown in Table 2 ▶.

## 3. Results   

### 3.1. Fragment screen against *Tb*NMT by NMR spectroscopy   

A diverse fragment library consisting of 652 molecules (mean molecular mass 187 Da, mean heavy-atom count 13.3) was screened against *Tb*NMT using ligand-observed NMR spectroscopy methods incorporating STD, water-LOGSY and CPMG experiments. The known ligand DDD85646 was used as a competitor molecule to identify molecules that interact with the protein within the peptide-binding groove. A total of 39 molecules (a hit rate of 6%) were identified that showed binding to *Tb*NMT and competed with DDD85646 in a single NMR experiment. This hit set was further classified depending on whether they showed binding and competition in all three NMR experiments (class I), two out of three experiments (class II) or only a single experiment (class III). 16 molecules were shown to be class I hits (2.5%) and 13 molecules (2.0%) were shown to be class II hits, with the remaining ten molecules (1.5%) designated as class III hits.

### 3.2. Structures of fragment molecules bound to *Lm*NMT   

A total of 28 molecules encompassing class I and II hits from the NMR screen were advanced to crystallographic experiments using the related *Lm*NMT as a structural surrogate. Soaking experiments were carried out for all 28 molecules and data sets were measured. Of the 28 ligand-soaking experiments, three data sets showed interpretable electron density corresponding to a bound fragment. One molecule, fragment **2**, was from the class I hit set, while the other two molecules, fragments **3** and **4**, were from the class II set.

#### 3.2.1. Structure of fragment **2** bound to *Lm*NMT   

**Figure 3:**
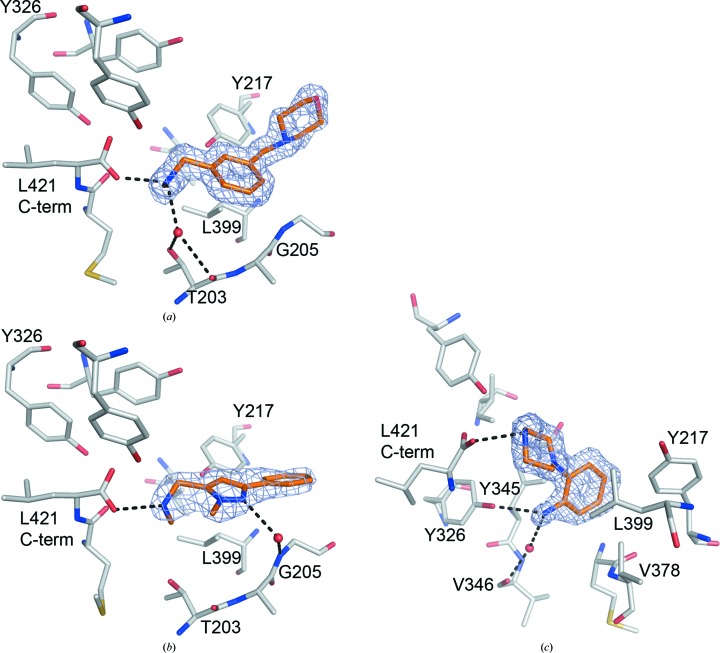
Binding modes of fragment hits bound to *Lm*NMT: (*a*) fragment **2**, (*b*) fragment **3** and (*c*) fragment **4**. Key protein residues (C atoms in grey) are shown in stick representation, as are the ligands (C atoms in gold). Key water molecules are shown as red spheres and hydrogen-bond interactions as dashed black lines. Refined electron density (2*mF*_o_ − *DF*_c_) for the ligands is shown contoured at 1σ as a blue mesh. Key residues are labelled for clarity.

The structure of fragment **2** bound to *Lm*NMT–MCoA shows the ligand to occupy the peptide-binding site in close proximity to the catalytic centre around the C-terminal carboxylate, with a direct hydrogen-bond interaction between the carboxylate and the methylamino NH (Fig. 3 ▶ *a*). It is assumed that the N atom will be protonated owing to the pH of the crystallization buffer (pH 5.6) and the acidic micro-environment around the carboxylate; therefore, a second hydrogen bond can be formed to a tightly bound water molecule, which in turn is coordinated to the side-chain OH and backbone carbonyl of Thr203. No additional specific interactions between ligand and protein are present as the central aryl ring packs against the side chains of Tyr217 and Leu399 and the morpholino moiety is oriented towards the bulk solvent.

#### 3.2.2. Structure of fragment **3** bound to *Lm*NMT   

The ligand fragment **3** shares structural similarity with fragment **2** as it has a pendant methylamino moiety. The structure of this protein–ligand complex shows that the molecule binds in a similar orientation, with the amino group interacting with the C-terminal carboxylate *via* a single hydrogen bond (Fig. 3 ▶ *b*). A second water-mediated interaction is formed between the pyrazole NH and the backbone NH of Gly205.

#### 3.2.3. Structure of fragment **4** bound to *Lm*NMT   

The structure of fragment **4** bound to *Lm*NMT shows the ligand to bind in the peptide-binding groove close to the C-terminal carboxylate, which hydrogen-bonds to the piperazine moiety (Fig. 3 ▶ *c*). The aniline group lies in a hydrophobic cleft lined by the side chains of Tyr217, Tyr345 and Val378. The aniline amino group hydrogen-bonds to the side chain of Tyr326 and *via* a water molecule to the backbone carbonyl of Val346.

### 3.3. Biochemical and kinetic characterization of fragment hits confirmed by X-ray crystallography   

Biochemical inhibition data were generated for fragments **2**–**4** against *Tb*NMT and *Lm*NMT using a radiometric assay. Owing to the low molecular weight and limited complexity of the fragment molecules, the inhibitory activity was too low to generate accurate full dose-response curves; therefore, only the percentage inhibition at 0.5 m*M* is reported. In the case of *Tb*NMT fragments **2** and **4** showed limited inhibition (<50%), whereas fragment **3** showed a reasonable level of inhibition at 70%. For *Lm*NMT all three fragments showed a similar level of inhibition at around 50%.

Owing to the low potency of the fragment hits, kinetic binding characterization was carried out using BLI. An initial experiment was carried out in the absence of the cofactor myristoyl-CoA, showing fragments **2** and **3** to have weak affinity for *Tb*NMT (760 and 530 µ*M*, respectively), whereas fragment **4** showed a greater affinity by an order of magnitude (48 µ*M*). Despite the small size of fragments **2** and **3**, the calculated ligand efficiencies are still disappointing (<0.3), whereas for fragment **4** the ligand efficiency is higher (0.45). This pattern is repeated with *Lm*NMT; however, the measured affinities, and therefore the ligand efficiencies, are lower. The experiment was repeated in the presence of myristoyl-CoA, as it has been shown previously that NMT acts *via* a bi-bi mechanism and the affinity of DD85646 was higher in the presence of cofactor (Frearson *et al.*, 2010 ▶). For fragments **2** and **3** the affinity increased, typically around fivefold against both enzymes, resulting in good ligand efficiencies of >0.3; however, *K*_d_ values could not be determined for fragment **4** against either enzyme owing to complicated sensograms that did not display classical binding kinetics.

## 4. Discussion   

An overall hit rate of 6% was obtained from NMR screening, comparable with known ligandable protein targets (Chen & Hubbard, 2009 ▶). From our previous studies with *Tb*NMT we had identified key hotspots within the peptide-binding groove, specifically the environs of the catalytic site around the C-terminal carboxylate. All three fragment hits interact with the C-terminal carboxylate through a basic N atom. Despite multiple attempts *via* soaking and co-crystallization, only three protein–ligand complexes were obtained from the set of 28 molecules identified as class I or II hits from the NMR screen. This low confirmation rate may be explained by the use of *Lm*NMT as a structural surrogate for *Tb*NMT, a protein that has not been crystallized to date. Kinetic binding data obtained for fragments **2**–**4** against *Tb*NMT and *Lm*NMT showed that the molecules have a lower affinity for *Lm*NMT, the structural surrogate. Despite the high sequence similarity across the whole protein and 94% sequence conservation within the active sites, we have observed a surprising degree of selectivity to be shown by molecules when assayed against NMTs from multiple kinetoplastid species (unpublished data), the origin of which remains elusive. It is postulated that the high level of conformational plasticity within the peptide-binding groove plays a major role in the selectivity profiles.

**Figure 4:**
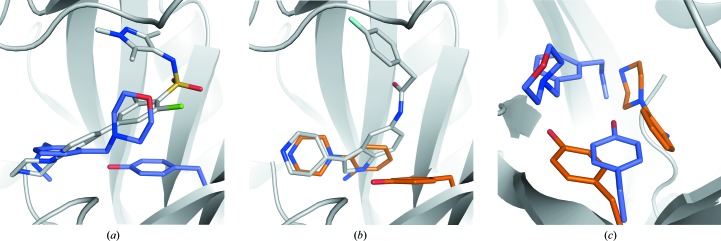
Comparison of fragment-binding modes to known NMT ligands. (*a*) Fragment **2** (C atoms in slate) overlaid with a pyrazole sulfonamide ligand (C atoms in grey) bound to *Lm*NMT (PDB entry 2wsa). (*b*) Fragment **4** (C atoms in gold) overlaid with a pyridinylindole ligand (C atoms in grey) bound to *Lm*NMT (PDB entry 4cgn). (*c*) Side-chain movement of Tyr217 in the complexes with fragment **4** (C atoms in gold) and fragment **2** (C atoms in slate).

To date, 19 structures of NMT from various species in complex with peptide-competitive ligands have been deposited in the Protein Data Bank. Despite the ligands representing multiple chemical series, two gross binding modes are observed depending upon the position of the Tyr side chain (Tyr217 in *Lm*NMT) that lies at the base of the peptide-binding groove. One binding mode, exemplified by benzofuran (Sogabe *et al.*, 2002 ▶) and recently pyridylindole ligands (Brannigan *et al.*, 2014 ▶), involves the ligand binding in a deep hydrophobic cleft delimited by the Tyr side chain. In the second binding mode, exemplified by aryl sulfonamides such as DDD85646 (Brand *et al.*, 2012 ▶; Frearson *et al.*, 2010 ▶), the Tyr side chain forms a hydrophobic platform upon which the hydrophobic moieties of the ligands stack. Fragments **2** and **3**, which contain a pendant methylamino moiety, show that the protein adopts the aryl sulfonamide conformation with Tyr217 forming a platform. Comparison of the fragment-binding modes with DDD85646 shows that the bicyclic systems of the fragments map to the piperazine–pyridine system (Fig. 4 ▶ *a*); however, the amide group of the fragments interacts directly with the C-terminal carboxylate, as opposed to the water-bridged interaction of the DDD85646 piperazine. In the case of fragment **4**, the piperazine group maps to the corresponding moiety of the piperazine–indole, with the aniline occupying a similar space in the binding cleft as the indole moiety (Fig. 4 ▶ *b*). The aniline amino group may present an additional vector for growing a new inhibitor into a previously uninvestigated pocket at the base of the active site. It is of interest that despite the small size and limited complexity of the fragment hits from this study, both binding-site configurations can be induced upon ligand binding, illustrating the conformational plasticity of the peptide-binding groove (Fig. 4 ▶ *c*). Fragment **4** showed limited enzyme inhibition in the biochemical assay, but however appeared to show the highest affinity for both NMT enzymes in the kinetic binding assay. In the presence of the cofactor myristoyl-CoA, complex sensograms were obtained using BLI that did not fit classical protein–ligand binding; therefore, further studies will be required to discover the origin of this effect.

In FBLD, fragment merging, combining moieties from multiple fragments or larger elaborated ligands, is a powerful technique for the rapid development of molecules which can address issues such as potency, selectivity, stability, toxicity and novelty. The addition of new fragment protein–ligand complexes provides more information that can be used to develop new generations of NMT inhibitors.

## Supplementary Material

PDB reference: *N*-myristoyltransferase bound to myristoyl-CoA and fragments, 4ucm

PDB reference: 4ucn

PDB reference: 4ucp

## Figures and Tables

**Table 1:** Data-measurement and model-refinement statistics for the *Lm*NMT proteinligand complexes described in this work Values in parentheses are for the highest resolution shell.

	Fragment **2**	Fragment **3**	Fragment **4**
PDB code	4ucp	4ucm	4ucn
Data measurement			
Source	ID14-EH4, ESRF	ID14-EH4, ESRF	I04-1, DLS
Space group	*P*2_1_	*P*2_1_	*P*2_1_
Unit-cell parameters (, )	*a* = 48.5, *b* = 90.9, *c* = 53.5, = 114.0	*a* = 48.1, *b* = 91.2, *c* = 53.7, = 112.7	*a* = 47.3, *b* = 91.2, *c* = 53.0, = 112.3
Resolution ()	45.01.50 (1.551.50)	44.02.32 (2.402.32)	50.01.80 (1.831.80)
Observations	228190	55541	124936
Unique observations	67421	17931	37067
*R*_merge_ (%)	4.1 (48.6)	2.8 (7.5)	3.4 (8.8)
*I*/(*I*)	18 (2.5)	24 (10)	33 (12)
Completeness (%)	94.7 (90.9)	97.4 (87.7)	96.2 (97.1)
Multiplicity	3.4 (3.3)	3.1 (2.5)	3.4 (3.4)
Refinement statistics			
Resolution range ()	45.01.50	40.02.32	50.01.80
*R*_work_/*R*_free_ (%)	16.9/19.6	17.8/24.3	16.8/19.9
No. of non-H atoms			
Protein	3354	3354	3354
Cofactor	63	63	63
Ligand	16	15	13
Solvent	400	102	324
Mean *B* factor (^2^)			
Protein	19	41	17
Cofactor	13	35	15
Ligand	20	60	20
Solvent	31	42	31
R.m.s. deviations			
Bond lengths ()	0.013	0.008	0.008
Bond angles ()	1.44	1.077	1.11

**Table 2:** Enzyme-inhibition statistics and characterization of binding kinetics for fragment molecules and *Tb*NMT and *Lm*NMT Percentage inhibition (PI) values were obtained using a single fragment concentration of 0.5m*M*. *K*_d_ values were determined using biolayer interferometry in the absence and presence of the cofactor myristoyl-CoA (MYA). Ligand efficiency metrics (LE) were calculated from the calculated *K*_d_ values.

	*Tb*NMT					*Lm*NMT				
		MYA		+MYA			MYA		+MYA	
Fragment	PI (%)	*K*_d_ (*M*)	LE	*K*_d_ (*M*)	LE	PI (%)	*K*_d_ (*M*)	LE	*K*_d_ (*M*)	LE
**2**	36	760	0.26	90	0.34	55	980	0.25	200	0.31
**3**	70	530	0.30	200	0.33	52	700	0.28	180	0.34
**4**	12	48	0.45	ND	ND	53	98	0.42	ND	ND
